# ‘It pushed me back into the human race’: evaluative findings from a community Christmas event

**DOI:** 10.1111/hsc.12342

**Published:** 2016-03-09

**Authors:** Tracy Collins, Christine Kenney, Gabrielle Hesk

**Affiliations:** ^1^ School of Health Sciences The University of Salford Salford UK; ^2^ School of Nursing, Midwifery, Social Work and Social Sciences The University of Salford Salford UK

**Keywords:** Christmas, community, intergenerational relationships, loneliness, older people, volunteering

## Abstract

Many older people in Britain spend Christmas day alone. The Christmas period may be especially difficult for older people who are socially isolated, living with dementia or who have physical impairments, and may feel particularly marginalised at this time of year. This paper draws on evaluative research findings from a community Christmas event held in December 2014 at the University of Salford for older people and their carers who would be on their own on Christmas day. A multi‐method approach was employed, seven guests took part in semi‐structured interviews to explore their experiences and perceptions of the event, seven staff and student volunteers participated in a group interview to explore and discuss their participation in the event. Data collection took place during April and May 2015. Interview transcripts were subjected to thematic analysis. Three overarching themes were identified from the interviews: ‘reasons for participants attending the event’, ‘a different Christmas day: the impact on guests and volunteers’, and ‘learning, planning and moving forwards’. The findings illustrate that a range of people participated in the Christmas day event for a variety of reasons. The event itself had a positive impact, including the shared experience of social belonging, for all involved. There are tangible longer term benefits as a result of the event, such as ongoing contact between participants and the development of supportive networks in the local community.

**What is known about this topic**

- Christmas is a significant social celebration in Britain.
- Many older people are likely to be alone over the Christmas period.
- There is a lack of research concerning the impact of Christmas events.

**What this paper adds**

- Christmas events provide a sense of social belonging.
- Younger people as well as older people experience loneliness at this time of year.
- Participation leads to wider longer term community benefits.

## Introduction

The Royal Voluntary Service (RVS 2012) reports that many older people are likely to be alone over the Christmas period. This social exclusion may intensify feelings of loss and loneliness in later life (Collins 2014a), and be detrimental to health and well‐being (Shankar *et al*. 2015; Campaign to End Loneliness, n.d.).

Christmas celebrations are not only ‘ritual performances’ but also social resources as family and friends are brought together ‘routinely’ once a year, giving people the opportunity to reinforce reciprocal ties (Shordike & Pierce 2005, Collins 2013). However, the celebration may also have negative consequences, including family friction, financial burden and loneliness (Nursing Times, 2008; Collins 2014a).

Many older people experience multiple and parallel transitions associated with ageing and the life course, such as chronic illness and the loss of relationships and roles, which may limit their opportunities to engage in activities with others (Bernard *et al*. 2004). Social isolation may be experienced by older people over the Christmas period due to factors such as the geographic dispersion of kin, friction between family members, financial strain and limited physical mobility (Collins 2014a).

Loneliness and social isolation are predominantly associated with older adults, and often services that aim to alleviate these problems view older people as a homogenous group failing to engage individuals that are ‘harder to reach’ (Cattan *et al*. 2003). While some studies suggest that older people value interventions that cater to common interests and social backgrounds (Cattan *et al*. 2003), others suggest that having a range of different friendships may protect against loneliness (Stevens & van Tilburg 2000, Shankar *et al*. 2015) and help to manage transitions such as later life widowhood (Collins 2013). In addition, having a robust social network is thought to act as a buffer to conditions such as dementia and to compensate for mild cognitive impairment (Fratiglioni *et al*. 2000).

Reciprocity in social support is thought to be valued by older people, this is often facilitated by volunteers belonging to the same generation and culture (Cattan *et al*. 2003). Indeed, volunteering across the lifespan is thought to enhance feelings of life satisfaction particularly in later life (Van Willigen 2000). Volunteering and community engagement are also associated with the norms of reciprocity and trust associated with social capital (Glover 2004).

In addition to generational support, intergenerational exchanges are also thought to be mutually beneficial for older and younger people as they share learning and resources which facilitate community cohesion (Hatton‐Yeo & Ohsako 2000). However as noted earlier, the geographic dispersion of kin may lead to younger and older family members becoming ‘segregated’ which impacts on positive exchanges between generations (Kaplan 1997).

The University of Salford hosted a lunch for 15 guests (eight older women and seven older men) who joined staff and student volunteers on Christmas day 2014. Guests were invited via local networks and organisations, including Occupational Therapists and Age UK. In order to manage numbers and due to the University having an Institute for Dementia, the initial focus was on people living with dementia and their carers. However, invitations were opened out to other older people who would be on their own on Christmas day. Nine staff volunteers organised and attended the event along with 19 student volunteers. These volunteers also brought along family members including children, friends and pets on the day.

Although many local communities and organisations support Christmas events for older people who would otherwise be alone (Community Christmas, n.d.), there is a lack of research evidence to support their efficacy (Collins 2014b). This research addresses the following question: What are the experiences of guests and volunteers who participate in a community Christmas event?

### Aims of the project

- To evaluate a community Christmas event (lunch and the opportunity to socialise for older people who would be on their own on Christmas day).
- To explore the experiences of the guests and volunteers involved.

## Methodology

The research adopted a qualitative multi‐method approach (Silverman 2013) in order to explore the experiences and perceptions of the guests and volunteers involved in the Christmas event. Ethics approval was gained from the University College Ethics Panel and written informed consent was sought from participants by the researcher prior to fieldwork (Israel & Hay 2006).

All 15 guests were invited by letter to take part in semi‐structured interviews to explore their experiences and perceptions of the event. All staff and student volunteers were invited by letter to participate in a semi‐structured group interview to explore and discuss their participation in the event. Semi‐structured interviews were selected in order to explore the participants lived experiences and subjective meanings (Kvale & Brinkmann 2009) of the Christmas day event.

Seven guests (four women and three men aged 62–86 years) volunteered to take part in semi‐structured interviews to explore their experiences and perceptions of the event. Interviews were conducted in the guests’ homes for their convenience. One‐to‐one interviews were conducted with two of the guests who live alone. Two guests were interviewed together as they are husband and wife. Three guests were interviewed together, a husband, wife and their neighbour. One of the guests has dementia, she was interviewed with her husband and neighbour who are also her main carers.

Seven staff and student volunteers (six women and one man aged 23–58 years) agreed to take part in a focus group interview at the University to explore and discuss their participation in the event. Although group interviews offer the opportunity to analyse interactions between participants, the focus in this case remained on exploring the experiences and perceptions of the volunteers involved in the community Christmas event.

Data collection took place during April and May 2015. Individual interviews and the focus group interview were recorded and later transcribed. The transcripts were subjected to thematic analysis as it offers a practical and adaptable method for qualitative research data analysis (Braun & Clarke 2006). The NVivo 10 qualitative data analysis software was also used to assist in the analysis of the interview data (Bazeley 2007, Lewins & Silver 2007). To ensure trustworthiness (Braun & Clarke 2013), a colleague who was not part of the Christmas event undertook an independent analysis of the anonymised scripts to promote deeper reflection and analysis as recommended by Whalley‐Hammell (2002). The findings from the ‘one‐to‐one’ interviews and the focus group interview were amalgamated in the development of the thematic framework.

## Findings

Characteristics of the guests and volunteers who took part in the study are presented in Box 1. Three overarching themes with sub‐themes were identified from the interviews. These are outlined in Table 1.

Box 1 **Characteristics of the guests and volunteers who took part in the study**

**Table:** 

Guests interviewed: 7	Volunteers interviewed: 7
Female: 4	Female: 6
Male: 3	Male: 1
Age 60–69: 3	Age 20–29: 1
Age 70–79: 2	Age 30–39: 1
Age 80–89: 2	Age 40–49: 2
	Age 50–59: 3
White British: 7	White British: 5
	Black British (mixed race): 1
	Asian: 1

**Table 1:** Overarching themes and sub‐themes

Overarching themes	Sub‐themes
Reasons for participants attending the event	Stress, obligation, hard work and monotony Giving, doing and filling a gap Limited family, lack of friends and losses Being alone and lonely, feeling excluded
A different Christmas day: the impact on volunteers and guests	Meaning, a special day and memories of Christmas Reciprocity, a multi‐layered experience A different Christmas, a catalyst for change Social belonging Intergenerational relationships, feeling at home and valued
Learning, planning and moving forwards	Ongoing contact, building relationships and confidence Developing supportive networks Challenges and opportunities for future engagement Sustainability

### Reasons for participants attending the event

Many of the guests attended the event due to having a lack of family or having family members living out of the area which would have led to them being alone over the festive period:

There's only the two of us … because she's only got one brother now her family has all gone. My family don't live up here they live down South, there's only my brother and he lives down there so. (Male guest, aged 66 years)

Because normally there's no transport is there … double fare in a taxi and no buses are on … so being able to go on Christmas day was really good because otherwise you'd be stuck in not able to get out. (Female guest, aged 62 years)

My family is all in Australia. (Female guest, aged 80 years)

I was in the care home and they wanted to get as many people out for Christmas … I don't think I was ready to … travel [to family]. I don't think I was ready to be sociable … with other company [family]. I was glad to get home and get into my corner. (Female guest, aged 89 years)

For the majority of the volunteers being involved in the event offered an escape from the stress, monotony and hard work of a ‘normal’ Christmas day and provided the opportunity to be a part of something more meaningful:

For years I'd just taken for granted that me and my family are altogether and have got the presents and the food, you just take for granted what it is. To actually give back to someone else who doesn't have that and give them the opportunity to go somewhere and share with other people … was a big thing for me, and it was something that I have always wanted to do. (Female staff volunteer, aged 55 years)

Some of the younger volunteers as well as the older guests described the experience of feeling alone and lonely at this time of year:

Like I'm an only child, I don't really mind, Christmas day like we have said is *so family*, I've got a lot of cousins and things like that and you know *friends* I would consider family but they are with their real families so. So what would have been a lot of time kind of sitting on my own waiting for my mum and dad to do the tea or … it was *being around people* that was really nice … I appreciated that on that specific day that I wasn't, I wouldn't be on my own but, but yeah a bit of a spare part sort of thing. (Female student volunteer, aged 23 years)

### A different Christmas day: the impact on guests and volunteers

Many of the guests and volunteers talked about the Christmas event as feeling ‘real’ and ‘homely’, special memories of past Christmases were evoked and both generational and intergenerational relationships were fostered on the day:

Having the different age ranges, I mean there were even kiddies there, which was lovely you know, and one of the little girls came up and she made me a card … you know … God you don't know how important that is … *emotionally important* … that was really lovely. (Male guest, aged 66 years)

And the children made me a Christmas card … two little girls … made it for me, lovely. (Female guest, aged 89 years)

Reciprocal benefits, including experiencing a ‘deeper’ meaning of the event, were also apparent for some of the volunteers:

I knew we were doing something *good* and that we were giving some time but I only had a certain … layer of understanding of that and I knew I was getting something out of it for myself in terms of my children seeing that there are other people not as privileged as them. But it was when I went to pick the woman up and her partner or her friend and she said she had been up since five o'clock she had not been able to sleep because she was so excited and I just thought *God* the realisation of *how* important that was. (Female staff volunteer, aged 46 years)

Importantly, the event provided a sense of social belonging for people who would otherwise have felt excluded and lonely particularly at this time of year:

Being able to get out and meet other people … instead of just sort of sitting here looking at each other, you know [he] is at home most of the time looking after me. I sometimes feel guilty so … when we were invited it meant that we could both go out and he hasn't got to worry about looking after me because we are both there. Plus you are meeting other people. (Female guest, aged 62 years)

For some of the guests the event has been a catalyst for change having a wider impact on their daily lives and activities:

All these lovely people gave up their *Christmas day* for me and other people and that's what they were doing, they gave up … and it pushed me back into the human race … That changed, started to change my attitude … And it was because of that day … I am doing other things now, because of that, leading on from that … I became a lot more positive. (Male guest, aged 66 years)

The majority of the guests and volunteers talked about the positive aspects of the event; however, one of the guests discussed the feelings of loneliness he experienced once the event was over:

I walked in and I sat down and there was all these people … and I was quite … overwhelmed and in the end I didn't want it to finish because when I came home … I knew, this was the down bit the downside, I knew that I would come home on Christmas day to … nothing after having that couple of hours around people and *talking* to people, which I haven't done for two and a half years since I've lived here. (Male guest, aged 66 years)

### Learning, planning and moving forwards

Subsequent meetings following on from the event have helped to reinforce social relationships, build confidence and encourage engagement outside of the immediate group setting:

On Christmas day there were a couple of people there that I already knew … I forgot her name she goes to line dancing, I'm a president of the bowling club and she goes there line dancing … but she was there at that do … and her husband, I don't know which one of them has Alzheimer's but he used to be a taxi driver he used to work with me years ago. And they were there she was talking to me telling me about some club or something at … for people with dementia. (Male guest, aged 73 years)

However, a number of complex challenges and diverse opportunities arose when organising a Christmas event, these were identified by some of the volunteers involved:

One of the problems with people who want to volunteer, they'll get that *feeling in* late November early December and *a lot* want to go and work with the homeless … and they'll have needed a DBS check and they'll have needed training on the do's and don'ts … and they've just not got a chance to be able to do it, so that's another thing you are not inundated but you got a lot of volunteers that want to do that and actually you don't want to overwhelm your guests. (Female staff volunteer, aged 33 years)

Developing supportive networks across communities is essential to future planning and sustainability; some of the volunteers talked about what they could do to further promote a future Christmas event and engage potential guests:

It could be something like I'll go and visit the dementia cafes and put the word out, just easy things to do. (Male staff volunteer, aged 53 years)

## Discussion

The findings of this research illustrate that Christmas day continues to be an important and symbolic celebration in British society for many people (Shordike & Pierce 2005, Collins 2013). The experiences of the guests and volunteers, as revealed by the multi‐method approach adopted in this study, indicate that the Christmas event had a largely positive and powerful impact contributing to feelings of social belonging.

The majority of guests attended the event due to having a lack of family and friends, or having family who lived some distance away which would have resulted in them being alone on Christmas day (RVS, 2012; Collins 2014a). Guests appreciated being with people of different ages, and the presence of children and pets added to the event feeling genuine and homely. This suggests that while having common interests and social backgrounds may be important (Cattan *et al*. 2003), having a range of people from a variety of backgrounds promotes intergenerational relationships and different friendships which may protect against loneliness (Stevens & van Tilburg 2000, Shankar *et al*. 2015) and promote community cohesion (Hatton‐Yeo & Ohsako 2000).

The reciprocal nature of the event is also evident in the accounts of the volunteers who described a more ‘meaningful’ experience of Christmas which enhanced their feelings of life satisfaction in addition to the guests (Van Willigen 2000). Similarly, some of the younger volunteers described feelings of loneliness and social exclusion at this time of year in addition to the older guests who attended (Collins 2014a; Shankar *et al*. 2015, Campaign to End Loneliness, n.d.). This has important implications for addressing loneliness and facilitating social inclusion across the life course rather than focussing predominantly on later life.

The Christmas event has also led to subsequent meetings between guests and volunteers and plans for future sustainable community events. This illustrates that the social interaction facilitated by the event has fostered wider reciprocity and trust associated with social capital formation (Glover 2004). Although both guests and volunteers considered the event to be a success, the findings demonstrate inherent challenges and opportunities which also need to be taken into account. For example, although the event had the capacity to host 30 guests, only 15 attended on Christmas day, which resulted in the event having more volunteers than guests. This suggests that there is still work to be done in engaging individuals that are ‘harder to reach’ (Cattan *et al*. 2003). However, one of the benefits of having ample volunteers was increased flexibility on the day, for example some volunteers came for the first half of the event, while others came for the second half of the event. This meant that volunteers could also spend Christmas day with their own family and friends as well as participating in the event which is important at this time of year (Shordike & Pierce 2005, Collins 2013). In addition, one of the guests highlighted the contrast between the social interaction experienced at the event and the loneliness experienced on returning home afterwards. This further indicates the importance of fostering supportive networks and social capital (Glover 2004) which extends beyond organised events of this kind.

The findings of this evaluative research contribute to the much needed evidence base to support the efficacy and impact of Christmas events for older people who would otherwise be alone (Collins 2014b, Community Christmas n.d.).

## Conclusions

A range of people attended the event for a variety of reasons. The event itself had a positive impact, including the shared experience of social belonging, for both guests and volunteers. There are tangible longer term benefits as a result of the event, such as ongoing contact and the development of supportive networks.

### Limitations and recommendations for future research

As this is a small qualitative study, generalisations are limited. Although the data generated from this multi‐method study are rich and saturated, further research could be conducted to build on these findings, for example a larger longitudinal mixed methods study to examine the longer term impact of Christmas events on guests and volunteers. Further consideration could also be given to ‘harder to reach groups’ where the ethnicity and culture of the guests, those who attend such events and those who do not, inform future practice.

### Relevance of the project to policy and practice

The project is relevant to both policy makers and practitioners who work with older people, particularly those who have an interest in alleviating social isolation and loneliness and promoting social inclusion and well‐being in later life. It is envisaged that this evaluative research will inform and support future sustainable community engagement projects of this kind and hopefully generate future funding for stakeholders.
